# The clinical efficacy of integrated care in combination with vasopressin for cardiogenic shock induced by acute myocardial infarction: A randomized controlled study protocol

**DOI:** 10.1097/MD.0000000000028985

**Published:** 2022-05-06

**Authors:** Ling Xu, Qunxing Li, Delu Yin, Guangyu Song, Hongyan Wu

**Affiliations:** The First People's Hospital of Lianyungang, Department of Cardiovascular Medicine,No. 182 North Tongguan Road, Lianyungang 222001, Jiangsu Province,P.R. China.

**Keywords:** acute myocardial infarction, cardiogenic shock, clinical randomized controlled trials, integrated nursing, protocol, vasopressin

## Abstract

**Background:**

Cardiogenic shock (CS) is the most serious complication of acute myocardial infarction (AMI) with high mortality, and the conventional nursing mode can not meet the clinical needs. Studies have shown that integrated care model has advantages for critical and chronic diseases. However, there is no clinical study to evaluate the clinical efficacy of this nursing model on cardiogenic shock induced by acute myocardial infarction (CS-AMI).

**Methods:**

This is a prospective randomized controlled trial to study the clinical efficacy of integrated care combined with vasopressin in the treatment of CS-AMI. Participants will be randomized in a 1:1 ratio to receive integrated care combined with vasopressin in the treatment group and conventional care combined with vasopressin in the control group. The patients will be followed up for 3 months after systematic treatment. Observation indicators include: length of hospital stay, quality of life score, blood pressure level, and nursing satisfaction score. Finally, SPASS 20.0 software will be used for statistical analysis of the data.

**Discussion:**

This study will evaluate the clinical efficacy of integrated nursing combined with vasopressin in the treatment of CS-AMI. The results of this study will provide a reference for selecting appropriate nursing programs for CS-AMI patients.

**Trial registration:**

OSF Registration number: DOI 10.17605/OSF.IO/K8CN4

## 1 Introduction

Acute myocardial infarction (AMI) refers to a disease in which the coronary blood supply is drastically reduced or completely interrupted due to various reasons, resulting in severe and lasting acute ischemia leading to myocardial cell necrosis,^[1]^ and is the most serious manifestation of coronary artery disease.^[2]^ There are many complications of acute myocardial infarction, including arrhythmia, heart failure and shock, etc. Cardiogenic shock (CS) is the most serious complication, and the clinical mortality of patients with cardiogenic shock induced by acute myocardial infarction (CS-AMI) is more than 40%, which is also the main cause of clinical death.^[3]^

In addition to timely and effective treatment, it is necessary to carry out reasonable and scientific nursing intervention for patients in the process of rescue and treatment, which can improve the success rate of treatment, shorten the length of stay after treatment, and speed up the physical and psychological rehabilitation of patients.^[4,5]^ The content of conventional nursing is relatively single, and it cannot meet the needs of patients for medical treatment and nursing services, nor can it cooperate with the treatment to obtain the ideal treatment effect.^[6,7]^ Integrated nursing is a process that starts with patients ’ needs, results from patients ’ satisfaction, combines medical care and cooperation, mobilizes the enthusiasm of patients and their families, and finally overcomes diseases and completes medical services.^[8]^ At present, the integrated nursing model has been used in Parkinson's disease, advanced cancer, chronic kidney disease and other chronic difficult diseases, and has received positive feedback.^[9–11]^ Vasopressin mainly acts on peripheral vascular α receptors, thereby contracting blood vessels and increasing blood pressure, and plays an important role in maintaining hemodynamics after fluid resuscitation in patients with shock.^[12]^ Studies have shown that vasopressin is a reasonable first-line drug for restoring blood pressure in patients with cardiogenic shock.^[13]^ There is currently a lack of standard clinical studies to evaluate the effect of integrated nursing model combined with vasopressin on CS-AMI. This study will explore the efficacy and safety of integrated nursing combined with vasopressin in the treatment of CS-AMI through a prospective randomized controlled study.

## 2 Materials and methods

### 2.1 Study design

**Figure 1:**
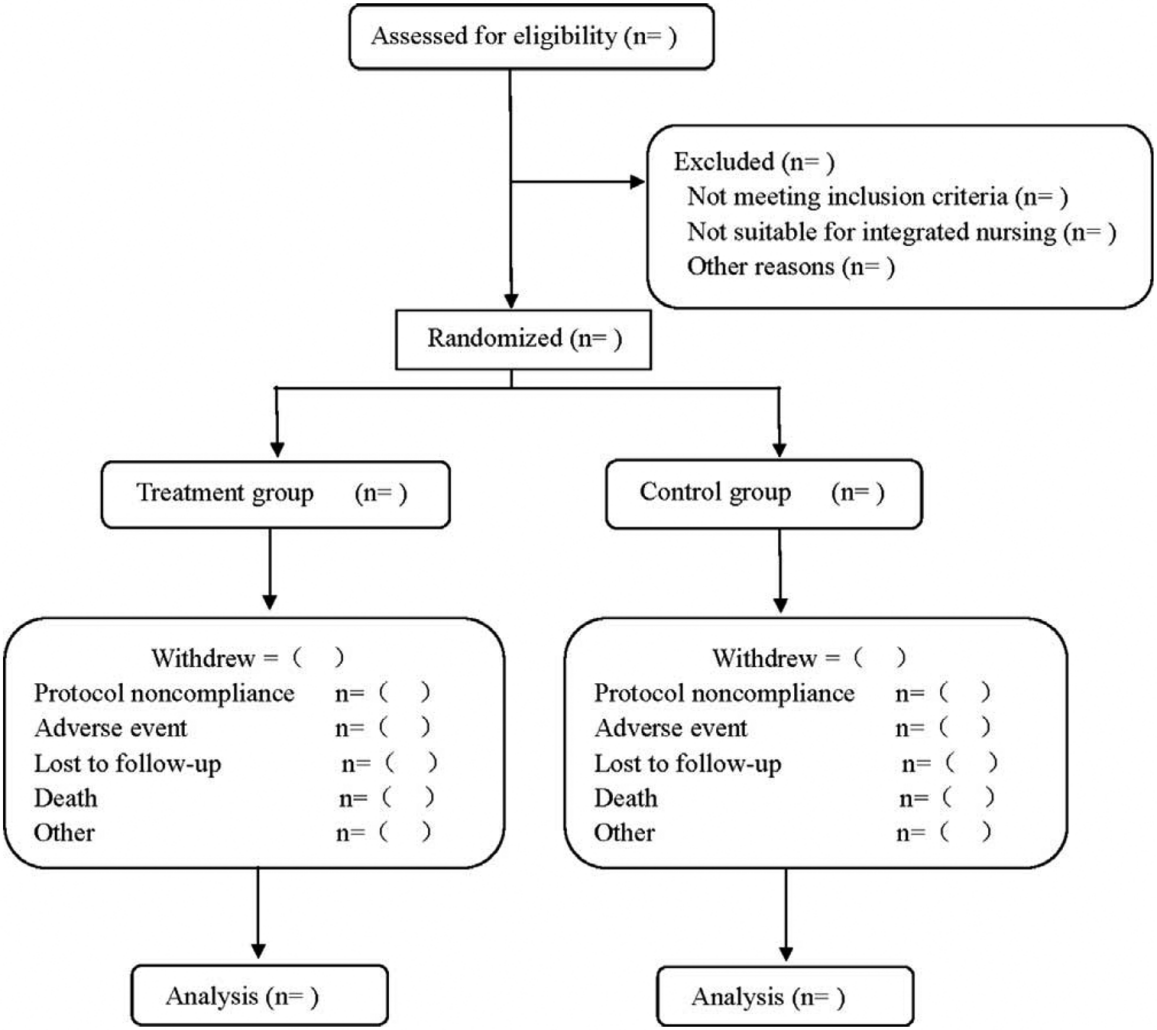
Flow diagram.

This is a prospective randomized controlled study of the clinical efficacy of integrated nursing combined with vasopressin in the treatment of CS-AMI. Patients meeting the requirements of initial screening will be randomly divided into treatment group and control group in a ratio of 1:1. The treatment group receives integrated nursing combined with vasopressin therapy, while the control group receives conventional nursing combined with vasopressin therapy. After the completion of systematic treatment, they will be followed up for 3 months. The flow diagram is shown in Figure 1.

### 2.2 Ethics and registration

This Research protocol will be conducted in accordance with the Declaration of Helsinki and Ethical Guidelines for Clinical Research. This Protocol will also be strictly in accordance with the latest Consolidated Standards of Reporting Trials (CONSORT 2017) and Standard Protocol Items: Recommendations for Interventional Trials (SPIRIT) 2013 Statement.This study has been approved by our Clinical Research Ethics Committee and registered with the Open Science Framework (OSF) (registration number: DOI 10.17605/OSF.IO/K8CN4). All patients and their families will sign a written informed consent form prior to the start of the study, and they can discontinue or withdraw from the study at any time during the study.

### 2.3 Patients

#### 2.3.1 Diagnostic criteria

Diagnostic criteria of CS-AMI^[14]^: (1) clinical tissue hypperfusion manifestations, such as cold and damp limbs, oliguria (< 30 mL/h) and/or delirium; (2) persistent hypotension, systolic blood pressure (SBP) < 80∼90 mm Hg, or mean arterial blood pressure more than 30 mm Hg lower than the original baseline measurement; (3) abnormal hemodynamics: cardiac index < 1.8L/(min m^2^) (untreated), or < 2.0∼2.2L /(min·m^2^)(treated), pulmonary capillary wedge pressure (PCWP) > 18 mm Hg, right end diastolic pressure > 10∼15 mm Hg.

#### 2.3.2 Inclusion criteria

1. Age ≥18;
2. Meet the diagnostic criteria of CS-AMI;
3. No serious or persistent mental illness (such as bipolar disorder, schizophrenia, etc.)
4. Subjects agree to participate in this study and sign informed consent.

#### 2.3.3 Exclusion criteria

1. Decreased blood pressure and shock caused by drugs, blood loss, pain, infection and other reasons;
2. patients with serious cardiac, hepatic and renal disorders;
3. patients with autoimmune diseases and malignant tumors;Those who have participated in or are currently participating in other clinical trials within the past 1 month.

### 2.4 Sample size

The sample size estimation of this study was based on the mean and standard deviation of the score of the MOS Item Short from health Survey of patients after treatment. Referring to the results of the preliminary experiment, the score of the treatment group was 70.76 ± 9.29, and that of the control group was 64.66 ± 10.76. set α=0.025, unilateral test, *β*=0.80. The PASS15.0 software calculated that 44 participants were required for each group, with an estimated dropout rate of 10%, and 49 patients would be enrolled in each group.

### 2.5 Randomization and blinding

Patients who meet the criteria will be randomly assigned to either the treatment group or the control group in a 1:1 ratio. Randomization procedures will be performed using SAS 9.3 software (SAS Institute, Cary, NC, USA) by independent statisticians not involved in trial implementation or statistical analysis using a centralized network-based randomization tool. The research assistant will enter patient information on a tablet computer and be assigned a random number to complete the random assignment. Due to the particularity of the intervention, the distribution results are known to the patients and the principal investigator, but not to the assistant and statistician responsible for data collection and analysis throughout the study.

### 2.6 Interventions

According to the recommendations of American Heart Association^[15]^ and European Society of Cardiology^[16]^ for the management of CS-AMI patients, both groups will receive blood volume replacement, oxygen, acidosis and electrolyte disturbance correction, vasoactive drug therapy, thrombolytic therapy, and intra-aortic balloon counterpulsation, etc. Norepinephrine will act as vasopressin to maintain blood pressure after fluid resuscitation.

The treatment group will adopt an integrated nursing program, the specific contents are as follows: (1) Establish a medical assistance group consisting of 1 medical team leader, 2 attending doctors, 1 nursing team leader and 6 responsible nurses on shifts, formulate post responsibilities, and the group is responsible for the overall management of patients. (2) Doctors and nurses make ward rounds together to encourage patients and their families to actively participate in diagnosis and treatment activities. On the one hand, the responsible nurse closely observe the patient's condition dynamics, timely inform the doctor in charge of the condition; On the other hand, patients and their families are encouraged to take the initiative to feedback their feelings during the diagnosis and treatment activities, so that medical staff can timely understand the changes in the condition and revise the diagnosis and treatment plan. (3) Revise and improve the treatment, nursing and rehabilitation plan of patients before discharge, and guide patients and their families to master it. (4) The responsible nurse is responsible for telephone return visit of patients after discharge, which includes patients’ condition, self-care, diet, rehabilitation and exercise, gives guidance, reminds patients to return visit on time, and improves the return visit record.

The control group adopted routine nursing program, including daily nursing health education and discharge guidance.

### 2.7 Outcomes

1. Length of hospital stay: defined as the time from the beginning of the study to the stabilization of the patient's condition and discharge.
2. Quality of life assessment: the MOS Item Short from health Survey.
3. Average blood pressure level: the average blood pressure of the patients in the morning, middle and night will be taken as the average blood pressure level of the day.
4. Nursing satisfaction evaluation: patients or family members make evaluation on the nursing process. The evaluation content includes rescue process, nurse-patient communication, service attitude and overall evaluation. The scoring standard for each item is unsatisfactory (0 points), general (1 points), satisfied (2 points) and very satisfied (3 points).

Two research assistants will assess the patients’ quality of life and blood pressure at baseline and discharge, record the length of hospital stay, and collect the patient's nursing satisfaction score at discharge. At the end of the first and third months of follow-up, blood pressure and quality of life assessment data will be collected by the study assistants either in the clinic or by telephone (patients unable to attend the clinic for objective reasons). Results of the randomization are unknown for the 2 study assistants.

### 2.8 Data management and quality control

To ensure the reliability of the data, all patients’ personal information will be collected and stored in a separate storage room limited to the researchers of the research team and must be accessed by two or more members to protect pre-, during-and post-trial confidentiality. All information about the patient will not be disclosed and transmitted without the patient's written permission.

### 2.9 Statistical analysis

The collected data will be statistically analyzed by SPSS 20.0 software. Chi-square test will be used for counting data. Mean ± standard deviation ($\bar{x}$ ± S) will be used for measurement data, and independent sample *t* test will be used for normal distribution, while Mann-Whitney *U* test will be used for skewness distribution. The difference will be considered statistically significant when *P* < 0.05.

## 3 Discussion

Patients with acute myocardial infarction complicated with cardiogenic shock are critically ill and need timely and effective treatment in a short time. Conventional nursing content is relatively single, and can not meet the needs of patients for medical treatment and nursing services, and can not cooperate with the treatment to obtain the ideal treatment effect.^[17,18]^ Therefore, the comprehensive nursing mode should be actively explored.

Through the implementation of the integrated model of medical care and patient, medical care timely, effectively and comprehensively assess, diagnose and care for patients, so that they can timely obtain effective treatment plan; Patients participate in treatment and nursing decisions to enhance their treatment confidence, thus promoting the improvement and control of the disease.^[19]^ When the body is in shock, vasopressin can increase blood pressure through antidiuretic and vasoconstrictor effects.^[20]^ Vasopressin only plays a role of boosting blood pressure by directly constricting blood vessels, but does not increase heart rate and cardiac oxygen consumption, especially for patients with arrhythmia and cardiac insufficiency.^[21]^ This study aims to explore the clinical efficacy of integrated nursing combined with vasopressin in the treatment of CS-AMI in a prospective randomized controlled trial.

There are also some shortcomings in this study. Firstly, due to the influence of intervention methods, this study cannot achieve double-blindness, which may lead to bias in research results. Secondly, this study belongs to a single-center study, patients included in the study may exist regionalization; finally, the family atmosphere of different patients after discharge may affect the psychology of patients, which may affect the follow-up results of the study.

## Author contributions

**Conceptualization:** Ling Xu and Qunxing Li

**Data curation:** Ling Xu and Delu Yin

**Formal analysis:** Ling Xu and Guangyu Song

**Funding acquisition:** Hongyan Wu

**Software:** Ling Xu and Guangyu Song

**Supervision:** Delu Yin and Guangyu Song

**Writing - original draft:** Ling Xu and Delu Yin

**Writing - review & editing:** Ling Xu and Guangyu Song
